# Supplementary material for: HIVprotI: an integrated web based platform for prediction and design of HIV proteins inhibitors
Source: J Cheminform. 2018 Mar 9;10:12. doi: 10.1186/s13321-018-0266-y (PMC5845081; doi:10.1186/s13321-018-0266-y)
Supplement: Supplementary file 2 — Additional file 2. Source code of HIVProtI web server. [file 13321_2018_266_MOESM2_ESM.zip › HIVprotI_Source-code/hivprotI/weka-3-6-6/doc/weka/gui/JTableHelper.html]

JTableHelper


---


|  |  |  |
| --- | --- | --- |
| **Overview** \| **Package** \| **Class** \| **Tree** \| **Deprecated** \| **Index** \| **Help** \| **Weka's home** | |  |
| **PREV CLASS**   **NEXT CLASS** | **FRAMES**    **NO FRAMES**     **All Classes** |  |
| SUMMARY: NESTED \| FIELD \| CONSTR \| METHOD | DETAIL: FIELD \| CONSTR \| METHOD |  |


---


## weka.gui Class JTableHelper

```
java.lang.Object
  weka.gui.JTableHelper
```

---

``` public class JTableHelper extends java.lang.Object ```

A helper class for JTable, e.g. calculating the optimal colwidth.

**Version:**
:   $Revision: 7059 $

**Author:**
:   FracPete (fracpete at waikato dot ac dot nz)

---

**Constructor Summary**

`JTableHelper(javax.swing.JTable jtable)`
  
          initializes the object


| **Method Summary** | |
| --- | --- |
| `int` | `calcColumnWidth(int col)`             calcs the optimal column width of the given column |
| `static int` | `calcColumnWidth(javax.swing.JTable table, int col)`             Calculates the optimal width for the column of the given table. |
| `int` | `calcHeaderWidth(int col)`             calcs the optimal header width of the given column |
| `static int` | `calcHeaderWidth(javax.swing.JTable table, int col)`             Calculates the optimal width for the header of the given table. |
| `javax.swing.JTable` | `getJTable()`             returns the JTable |
| `void` | `scrollToVisible(int row, int col)`             Assumes table is contained in a JScrollPane. |
| `static void` | `scrollToVisible(javax.swing.JTable table, int row, int col)`             Assumes table is contained in a JScrollPane. |
| `void` | `setOptimalColumnWidth()`             sets the optimal column width for all columns |
| `void` | `setOptimalColumnWidth(int col)`             sets the optimal column width for the given column |
| `static void` | `setOptimalColumnWidth(javax.swing.JTable jtable)`             sets the optimal column width for alls column if the given table |
| `static void` | `setOptimalColumnWidth(javax.swing.JTable jtable, int col)`             sets the optimal column width for the given column |
| `void` | `setOptimalHeaderWidth()`             sets the optimal header width for all columns |
| `void` | `setOptimalHeaderWidth(int col)`             sets the optimal header width for the given column |
| `static void` | `setOptimalHeaderWidth(javax.swing.JTable jtable)`             sets the optimal header width for alls column if the given table |
| `static void` | `setOptimalHeaderWidth(javax.swing.JTable jtable, int col)`             sets the optimal header width for the given column |

**Methods inherited from class java.lang.Object**

`equals, getClass, hashCode, notify, notifyAll, toString, wait, wait, wait`

**Constructor Detail**

### JTableHelper

```
public JTableHelper(javax.swing.JTable jtable)
```

:   initializes the object


**Method Detail**

### getJTable

```
public javax.swing.JTable getJTable()
```

:   returns the JTable

---


### calcColumnWidth

```
public int calcColumnWidth(int col)
```

:   calcs the optimal column width of the given column

---


### calcColumnWidth

```
public static int calcColumnWidth(javax.swing.JTable table,
                                  int col)
```

:   Calculates the optimal width for the column of the given table. The
    calculation is based on the preferred width of the header and cell
    renderer.
      
    Taken from the newsgoup de.comp.lang.java with some modifications.  
    Taken from FOPPS/EnhancedTable - http://fopps.sourceforge.net/  

    :   **Parameters:**: `table` - the table to calculate the column width: `col` - the column to calculate the widths **Returns:**: the width, -1 if error

---


### calcHeaderWidth

```
public int calcHeaderWidth(int col)
```

:   calcs the optimal header width of the given column

---


### calcHeaderWidth

```
public static int calcHeaderWidth(javax.swing.JTable table,
                                  int col)
```

:   Calculates the optimal width for the header of the given table. The
    calculation is based on the preferred width of the header renderer.

    :   **Parameters:**: `table` - the table to calculate the column width: `col` - the column to calculate the widths **Returns:**: the width, -1 if error

---


### setOptimalColumnWidth

```
public void setOptimalColumnWidth(int col)
```

:   sets the optimal column width for the given column

---


### setOptimalColumnWidth

```
public static void setOptimalColumnWidth(javax.swing.JTable jtable,
                                         int col)
```

:   sets the optimal column width for the given column

---


### setOptimalColumnWidth

```
public void setOptimalColumnWidth()
```

:   sets the optimal column width for all columns

---


### setOptimalColumnWidth

```
public static void setOptimalColumnWidth(javax.swing.JTable jtable)
```

:   sets the optimal column width for alls column if the given table

---


### setOptimalHeaderWidth

```
public void setOptimalHeaderWidth(int col)
```

:   sets the optimal header width for the given column

---


### setOptimalHeaderWidth

```
public static void setOptimalHeaderWidth(javax.swing.JTable jtable,
                                         int col)
```

:   sets the optimal header width for the given column

---


### setOptimalHeaderWidth

```
public void setOptimalHeaderWidth()
```

:   sets the optimal header width for all columns

---


### setOptimalHeaderWidth

```
public static void setOptimalHeaderWidth(javax.swing.JTable jtable)
```

:   sets the optimal header width for alls column if the given table

---


### scrollToVisible

```
public void scrollToVisible(int row,
                            int col)
```

:   Assumes table is contained in a JScrollPane.
    Scrolls the cell (rowIndex, vColIndex) so that it is visible
    within the viewport.

---


### scrollToVisible

```
public static void scrollToVisible(javax.swing.JTable table,
                                   int row,
                                   int col)
```

:   Assumes table is contained in a JScrollPane.
    Scrolls the cell (rowIndex, vColIndex) so that it is visible
    within the viewport.


---


|  |  |  |
| --- | --- | --- |
| **Overview** \| **Package** \| **Class** \| **Tree** \| **Deprecated** \| **Index** \| **Help** \| **Weka's home** | |  |
| **PREV CLASS**   **NEXT CLASS** | **FRAMES**    **NO FRAMES**     **All Classes** |  |
| SUMMARY: NESTED \| FIELD \| CONSTR \| METHOD | DETAIL: FIELD \| CONSTR \| METHOD |  |


---
